# Decay stages of wood and associated fungal communities characterise diversity–decomposition relationships

**DOI:** 10.1038/s41598-021-88580-2

**Published:** 2021-04-26

**Authors:** Yu Fukasawa, Kimiyo Matsukura

**Affiliations:** 1 Graduate School of Agricultural Science, Tohoku University, 232-3 Yomogida, Naruko, Osaki, Miyagi 989-6711 Japan; 2 Sado Island Center for Ecological Sustainability, Niigata University, 94-2 Koda, Sado, Niigata 952-2206 Japan

**Keywords:** Biodiversity, Microbial ecology

## Abstract

The biodiversity–ecosystem function relationship is a central topic in ecology. Fungi are the dominant decomposers of organic plant material in terrestrial ecosystems and display tremendous species diversity. However, little is known about the fungal diversity–decomposition relationship. We evaluated fungal community assemblies and substrate quality in different stages of wood decay to assess the relationships between fungal species richness and weight loss of wood substrate under laboratory conditions. Wood-inhabiting fungal communities in the early and late stages of pine log decomposition were used as a model. Colonisation with certain species prior to inoculation with other species resulted in four-fold differences in fungal species richness and up to tenfold differences in the rate of wood substrate decomposition in both early- and late-decaying fungal communities. Differences in wood substrate quality had a significant impact on species richness and weight loss of wood and the relationships between the two, which were negative or neutral. Late communities showed significantly negative species richness–decay relationships in wood at all decay stages, whereas negative relationships in early communities were significant only in the intermediate decay stage. Our results suggest that changes in fungal communities and wood quality during wood decomposition affect the fungal diversity–decomposition relationship.

## Introduction

Fungi are the dominant decomposers of organic plant material in terrestrial ecosystems and directly affect global carbon and nutrient dynamics^1–3^. Despite their importance, our understanding of the relationships between fungal species diversity and decomposition still lags behind that of plant diversity–productivity relationships^4,5^. Previous field studies have reported positive, neutral and negative relationships between fungal species richness and decomposition rates, which are measured as a decrease in the weight or density of plant materials^6–10^. In contrast, laboratory studies carried out under controlled conditions have reported negative relationships between fungal species richness and weight loss of plant materials^11,12^. To explain this discrepancy, a better understanding of the mechanisms driving fungal diversity–decomposition relationships is required.

A complicating factor encountered when studying fungal species diversity–decomposition relationships on natural resources is successional changes in fungal communities and substrate quality during decomposition. Fungal succession during wood decomposition has been well described^13,14^. Living wood already has latent fungal inhabitants^15^, and a portion of these latent fungi become primary colonisers immediately after tree death. These primary colonisers utilise both the labile components of wood, such as sap sugars, and recalcitrant lignocellulose^16,17^. Secondary colonisers arrive via airborne spores, competitively replace the primary colonisers and take part in the intermediate stages of decomposition. These secondary colonisers decay lignocellulose intensively^18,19^. Late colonisers, such as cord-forming fungi from soil, are most competitive; they become established after the secondary colonisers and complete the final stages of wood decomposition^13^. The competitive ability of fungi is generally greater in communities at later stages of wood decomposition than at earlier stages^20^. In addition, fungal species associated with disturbances and symbiotic associations with tree roots dominate the final stages of wood decomposition^20–22^. Overall, fungal species composition completely changes during wood decomposition (a phenomenon known as substrate succession)^23^. Fungal species richness is known to increase with decay progresses and peak in the most decayed substrates^21,22^.

Additionally, wood quality changes during wood decomposition. In general, wood density decreases and water content increases greatly as decay progresses^24^. Furthermore, delignification of wood by white rot fungi and holocellulose-selective decomposition by brown rot fungi both alter wood chemical composition in the intermediate stages of decay, leaving the recalcitrant components of wood, such as lignin, to accumulate in the later stages. These physicochemical changes affect fungal competition, and community development^25,26^ and wood decay abilities^27,28^, and thus should also be studied to reveal their impact on fungal diversity–decomposition relationships. In fact, van der Wal et al.^7^ reported from field monitoring of dead oak stumps that the relationship between fungal species richness and wood weight loss became positive in later stages of decay, whereas no relationships were observed in earlier stages. Laboratory studies in which fungal communities and resource quality are carefully controlled are needed to clarify the complex fungal diversity–decomposition relationships on natural resources.

The effects of interspecific fungal interactions on wood decomposition have been explained by a combination of three mechanisms^20^: selection, resource partitioning, and facilitation, all of which produce a positive relationship between fungal interactions and decomposition. Such mechanisms are analogous to hypotheses about biodiversity–productivity relationships, which have been studied mainly in plant communities^4,5^. In contrast, recent studies of wood decay fungi emphasise the importance of physiological changes, such as changes in carbon use efficiency (CUE), measured as the relative amount of biomass production per unit respiration rate (or decomposition rate)^29^. Such physiological changes might reflect competitive stress in fungal communities that show higher diversity^20,29^. Maynard et al.^29^ demonstrated that competitive fungal communities show negative relationships between fungal species richness and CUE, but these relationships were positive in non-competitive communities. However, a negative relationship between species richness and CUE might not directly result in a negative relationship between species richness and substrate weight loss due to differences between the two variables. Low CUE may retard substrate weight loss if its energetic cost slows down other physiological activities, such as the production of decay enzymes^30–32^, but it may accelerate substrate weight loss if a fungus activates decomposition to compensate for this energetic cost^25,33^. Although the respiration rate is thought to represent the instantaneous decomposition rate^28,34–36^, respiration is also associated with metabolic processes other than decay. Furthermore, carbon loss by leaching, which is another important part of decomposition^37^, could not be taken into account in previous respiration studies. Thus, diversity–decomposition relationships must be studied in more realistic situations using natural resources with substrate weight loss as a measure of decay.

**Figure 1:**
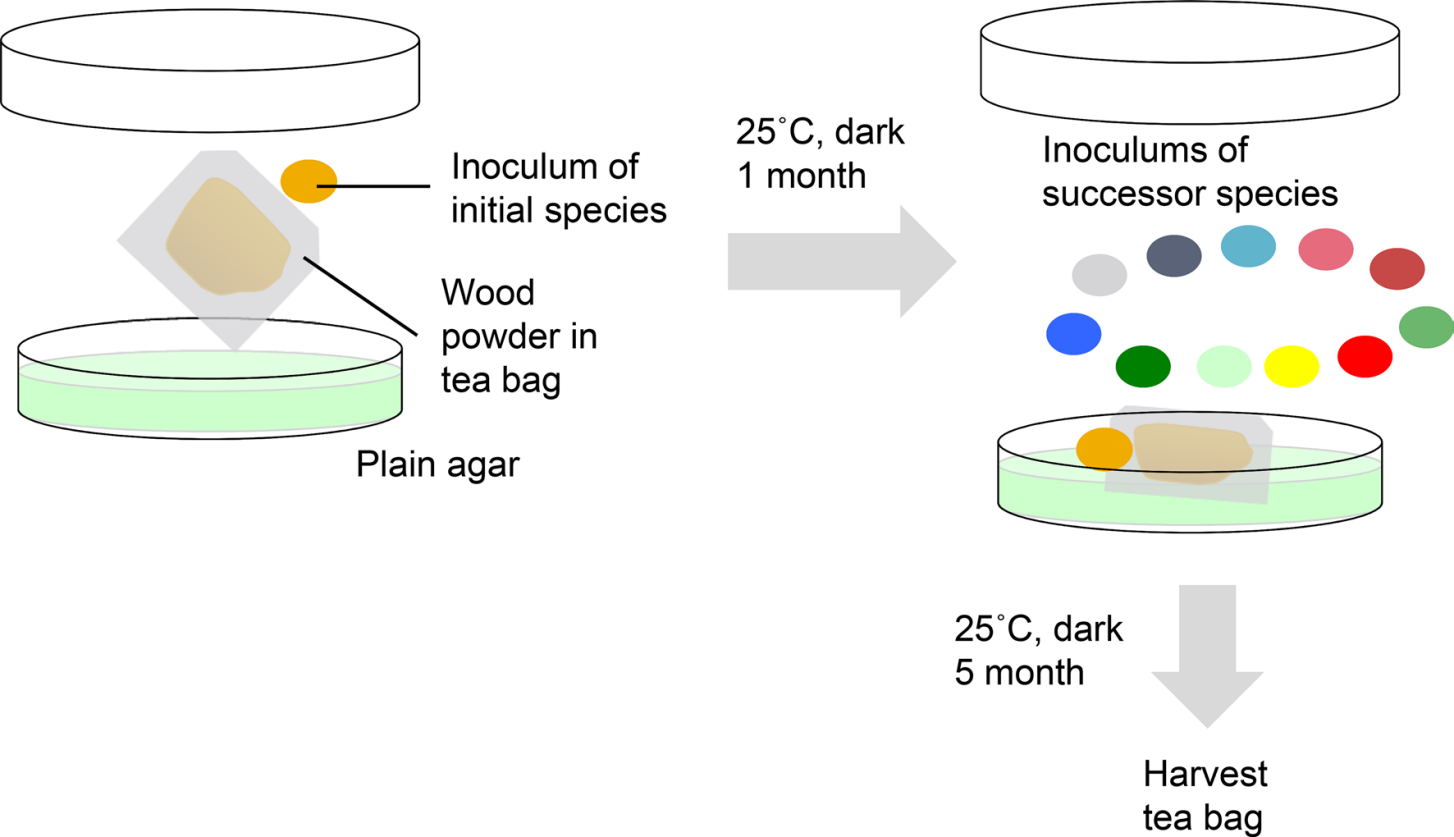
Schema of the experimental set up. Different colors of inocula indicates fungal species difference.

Communities of wood-inhabiting fungi have been studied thoroughly in terms of their interspecific interactions and succession as discussed above and are thus suitable for the study of diversity–decomposition relationships based on fungal interactions. In the present study, we define fungal diversity as the richness of fungal operational taxonomic units (OTUs) detected after the experiment and decomposition as the weight loss of the wood substrate. We used laboratory microcosms with full factorial combinations of fungal communities in early and late stages of wood decomposition (referred to as group E and L, respectively) along with wood substrates in various stages of decomposition to separately evaluate the effects of fungal community composition and wood substrate quality on fungal diversity–decomposition relationships (Table 1). We manipulated the assembly histories of the communities by selecting an initial species and several successor species for each microcosm (Table 1; Fig. 1) to induce variety in species diversity through the priority effect by incubation with the same set of species after the incubation period^12^. We used pine (*Pinus densiflora*) sapwood obtained from living pine trees and naturally decaying pine logs in intermediate and late decay stages as substrate with fungal communities obtained from pine deadwood as a model system because fungal community succession and wood physicochemical changes over time are well described in such systems^38–40^. We raised two working hypotheses: (1) Fungal communities in the early and late stages of wood decomposition differ in their competitive abilities and thus show different fungal diversity–decomposition relationships; and (2) Differences in the chemical qualities of wood in the early, intermediate, and late stages of decomposition affect fungal competition and resource use and thus produce different trends in fungal diversity–decomposition relationships.

**Table 1:** Fungal species used in the microcosm experiments.

Species name	Code	Phyllum	Decay type	NBRC^†^	DDBJ^¶^	Source
**Group E (early)**						
Armillaria cepistipes*	Armi	Basidio	White	110165	AB907593	W
Cryptoporus volvatus*	Cryp	Basidio	White	110166	LC100004	S
Fomitopsis pinicola*	Fomi	Basidio	Brown	110169	LC100006	S
Gloeophyllum sepiarium*	Gloe	Basidio	Brown	110172	LC100007	S
Helotiales sp. *	Helo	Asco	–	110173	AB907606	W
Leptographium lundbergii*	Lept	Asco	–	110179	AB907601	W
Mariannaea elegans*	Mari	Asco	–	110192	LC100008	W
*Phlebia livida*	–	Basidio	White	110185	AB907576	W
Phlebiopsis castanea*	Phle	Basidio	White	110168	LC100005	S
Resinicium bicolor*	Resi	Basidio	White	110186	AB907577	W
Trichaptum abietinum*	Tcha	Basidio	White	110190	AB907580	W
**Group L (late)**						
Gymnopilus liquiritiae*	Gymn	Basidio	White	110174	LC100009	S
*Hypochnicium eichleri*	–	Basidio	White	110176	AB907586	W
Kuehneromyces mutabilis*	Kueh	Basidio	White	110177	AB907596	W
Neolentinus suffrutescens*	Neol	Basidio	Brown	110181	LC100012	S
Penicillium lividum*	Peni	Asco	–	110182	LC100013	W
Phanerochaete velutina*	Phan	Basidio	White	110184	AB907604	W
Pholiota brunnescens*	Phol	Basidio	White	110175	LC100010	S
Rhinocladiella atrovirens*	Phin	Asco	–	110187	LC100014	W
*Scytinostroma odoratum*	–	Basidio	White	110188	AB907590	W
Sistotrema brinkmannii*	Sist	Basidio	White	110189	AB907592	W
Trichoderma harzianum*	Tder	Asco	–	–	LC100015	W
Umbelopsis isabellina*	Umbe	Muco	–	110180	LC100011	W

*W* deadwood, *S* sporocarp.

*Species selected as initial species.

^†^Culture strain number in Nite Biological Resource Center (NBRC).

^¶^Accession number in DNA data bank of Japan.

## Results

### Effects of initial species on fungal community

**Figure 2:**
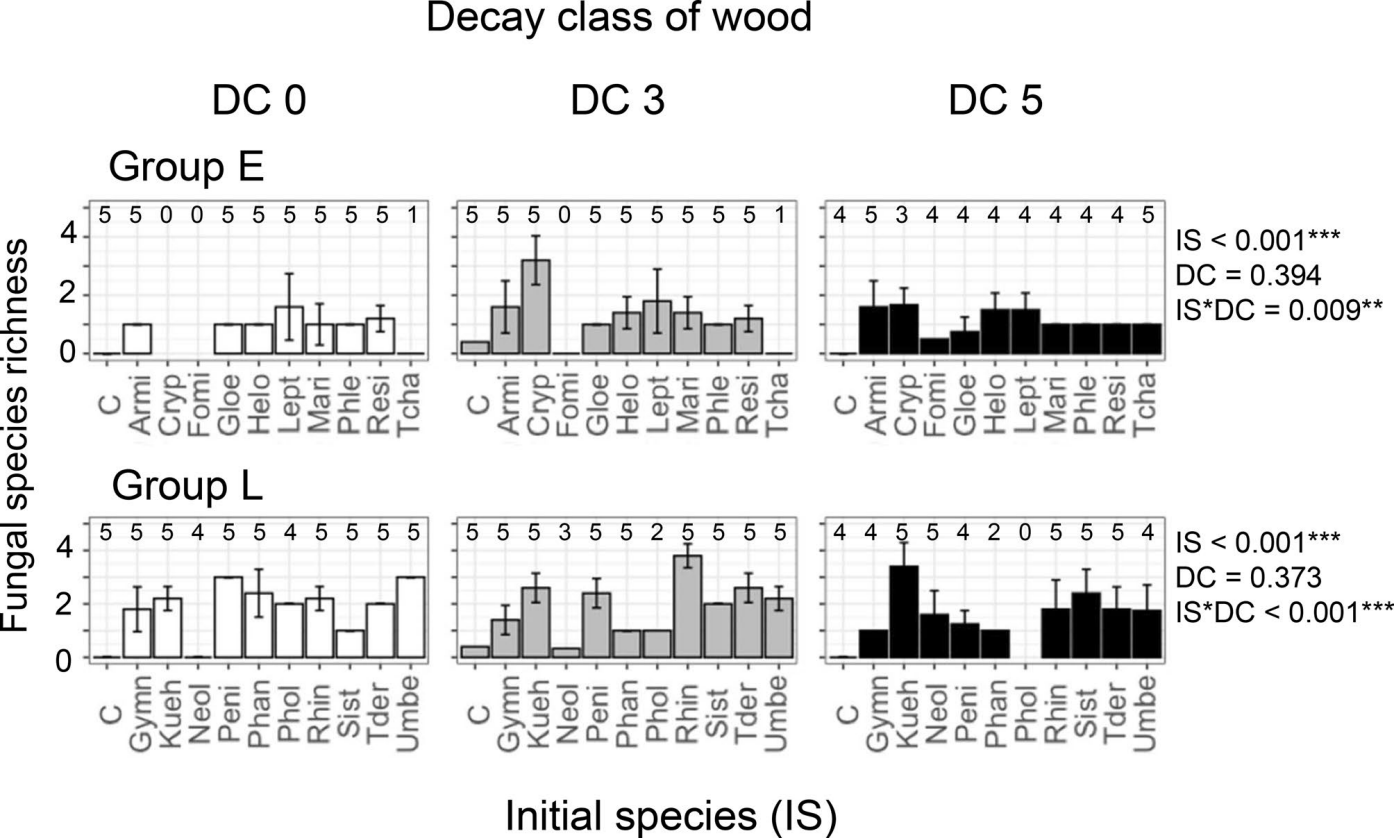
Effect of fungal colonization history on fungal species richness. Species richness (mean ± SD) was measured 5 months after all species were introduced. *P* values from ANOVAs are provided for effects of initial species (IS), decay class (DC), and their interactive effects (IS*DC). Asterisks indicate **P < 0.01 and ***P < 0.001. Codes for initial species are provided in Table 1. C, control (sterile agar plugs were introduced instead of fungi plugs). Numbers on the bars indicate replicate number of the sample. Color of the bars indicate DC of wood substrates: white, DC 0; grey, DC 3; black, DC 5.

After the filtering process and rarefaction of fungal DNA amplicon sequences, 24 fungal OTUs were detected after the experiments (Supplementary Table S1). Seven OTUs were found in each group (group E and L). Four species in group E (*Armillaria cepistipes*, *Fomitopsis pinicola*, *Leptographium lundbergii*, *Trichaptum abietinum*) and five species in group L (*Pholiota brunnescens*, *Kuehneromyces mutabilis*, *Neolentinus suffrutescens*, *Scytinostroma odoratum*, *Trichoderma harzianum*) were not detected. Even though the wood powder was sterilised with ethylene oxide gas before the experiment, ten fungal OTUs that were not used as inocula were detected (Supplementary Table S1). Regardless of whether these contaminant OTUs were excluded (Fig. 2) or included (Supplementary Fig. S1), manipulation of early assembly history during the initial month resulted in substantial variation in fungal species richness in both groups E and L, which persisted for at least 5 months. Fungal species richness differed by up to four-fold between early assembly history treatments across different decay classes (Fig. 2).

**Figure 3:**
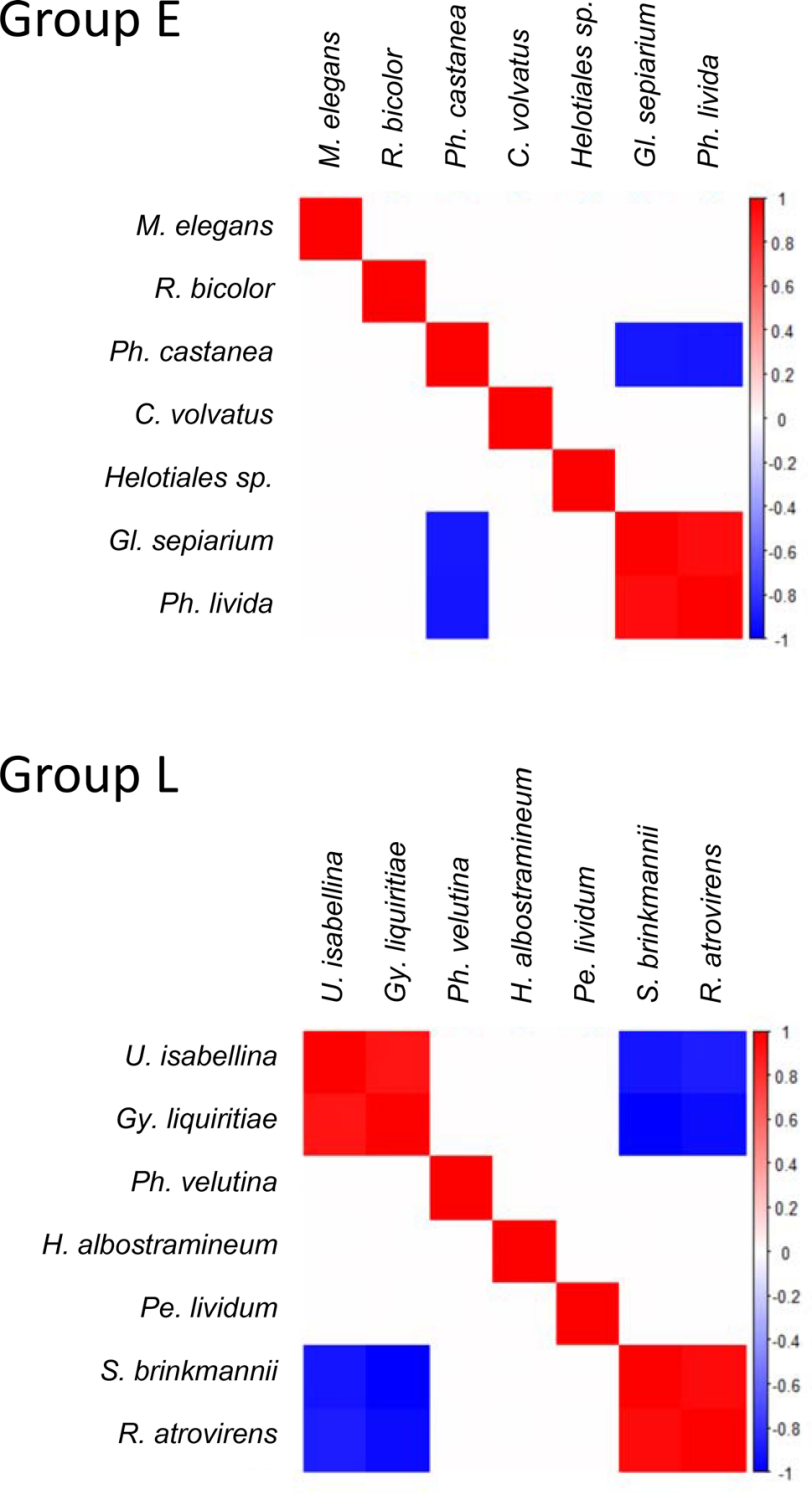
Association networks of fungal OTUs in groups E and L, according to sequence read number. Positive associations are displayed in red, and negative in blue. Only associations with a posterior probability above 95% are colored. OTUs were ordered to optimize visualization of association clusters.

Our data indicate that these differences in fungal communities resulted from complex interspecific interactions, with outcomes dependent on immigration history and substrate quality. Most of the species showed large relative abundances in microcosms in which the focal fungus was inoculated first as an initial species (Supplementary Fig. S2). In particular, *Phanerochaete velutina* (Phan), *Penicillium lividum* (Peni), and *Umbelopsis isabellina* (Umbe) in group L were detected almost exclusively from microcosms in which they were inoculated as initial species, regardless of substrate decay class. Similarly, *Phlebiopsis castanea* (Phle) and *Gloeophyllum sepiarium* (Gloe) in group E and *Gymnopilus liquiritiae* (Gymn) and *Sistotrema brinkmannii* (Sist) in group L showed the largest relative abundance in microcosms in which they were initial species. For other species, however, the results were highly dependent on the decay class (DC) of the substrates. Association networks of fungal species showed a positive association between *Phlebia livida* and *Gl. sepiarium* and negative associations between these two species and *Ph. castanea* in group E microcosms (Fig. 3). Two positive associations were detected in group L microcosms between *Gy. liquiritiae* and *U. isabellina*, and between *S. brinkmannii* and *Rhinocladiella atrovirens*, and four negative associations were detected among *S. brinkmannii*, *R. atrovirens*, *U. isabellina*, and *Gy. liquiritiae*.

### Effect of assembly history on wood decomposition

**Figure 4:**
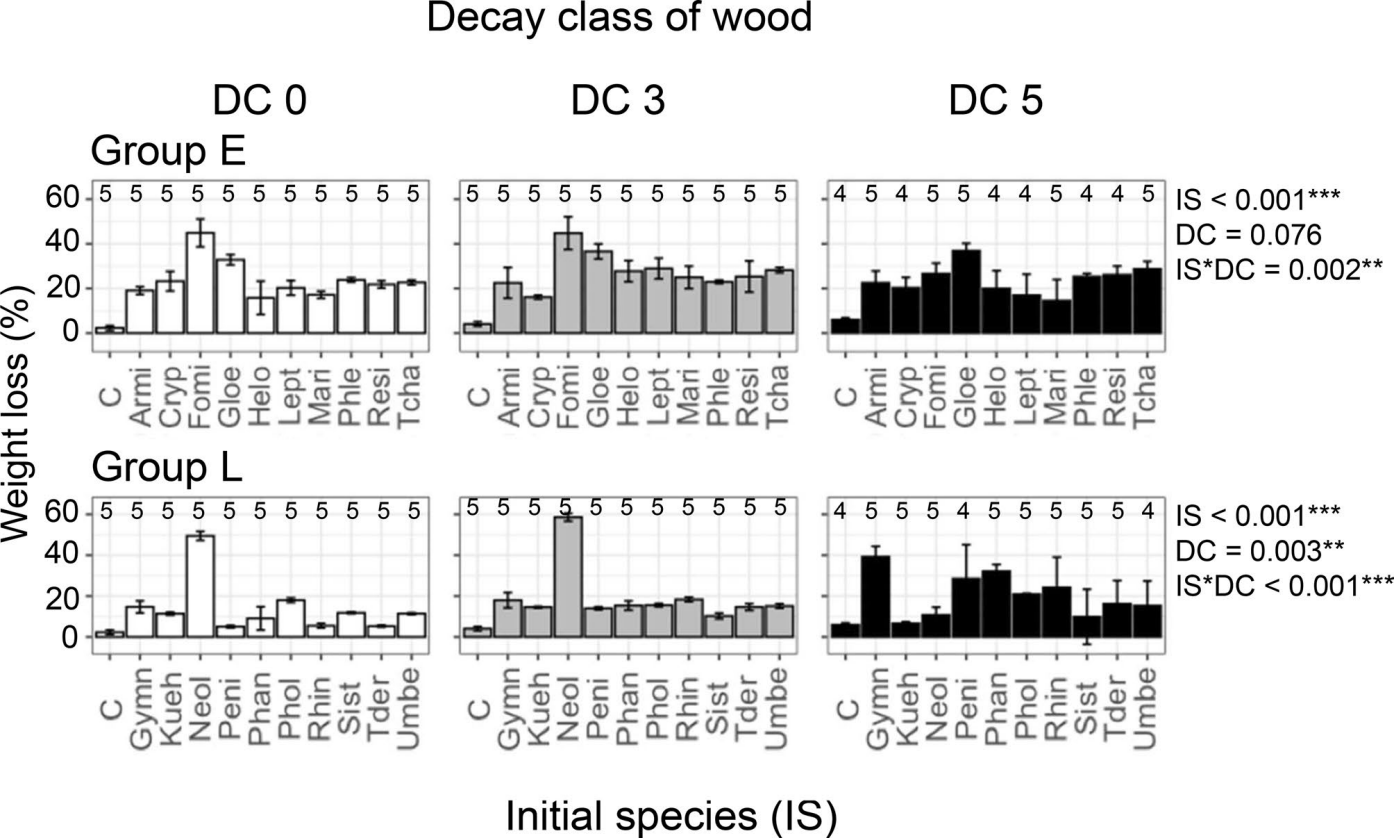
Effect of initial fungal species on wood decomposition 5 months after all species were introduced. Numbers on the bars indicate replicate number of the sample. *P* values and bar colors as in Fig. 2.

Assembly history also had striking effects on wood decomposition. We found over three-fold differences in group E and over tenfold differences in group L in wood weight loss among assembly history treatments (Fig. 4). The effects of wood decay class were not detected in group E, but were significant in group L. For example, weight loss in DC 5 wood in which *Gy. liquiritiae* (Gymn) and *Ph. velutina* (Phan) were introduced first was significantly greater than in DC 0 and DC 3 wood (Tukey-HSD, *adj.P* < 0.05). Similarly, weight loss in DC 5 wood in which *Pe. lividum* (Peni) and *R. atrovirens* (Rhin) were introduced first was significantly greater than loss in DC 0 wood (Tukey-HSD, *adj.P* < 0.05). Weight loss in DC 5 wood in which *Pholiota brunnescens* (Phol) was introduced first was significantly greater than in DC 3 wood (Tukey-HSD, *adj.P* < 0.05). In contrast, weight loss in DC 5 wood in which *Neolentinus suffrutescens* (Neol) and *Kuehneromyces mutabilis* (Kueh) were introduced first was significantly less than in DC 0 wood (Tukey-HSD, *adj.P* < 0.01). The contrasting effects of wood decay class depending on initial species were reflected in the significant effect of interaction between the initial species and wood decay class on wood decomposition. Control wood (inoculated with sterile agar plugs) of DC 0, 3, and 5 showed only 2.3 ± 1.1%, 4.1 ± 1.0%, and 6.0 ± 0.9% weight loss, respectively. Although contaminant OTUs dominated in control wood in terms of relative abundance (Supplementary Fig. S2), wood decomposition data indicated that the contribution of these OTUs to wood decomposition was limited (Fig. 4).

**Figure 5:**
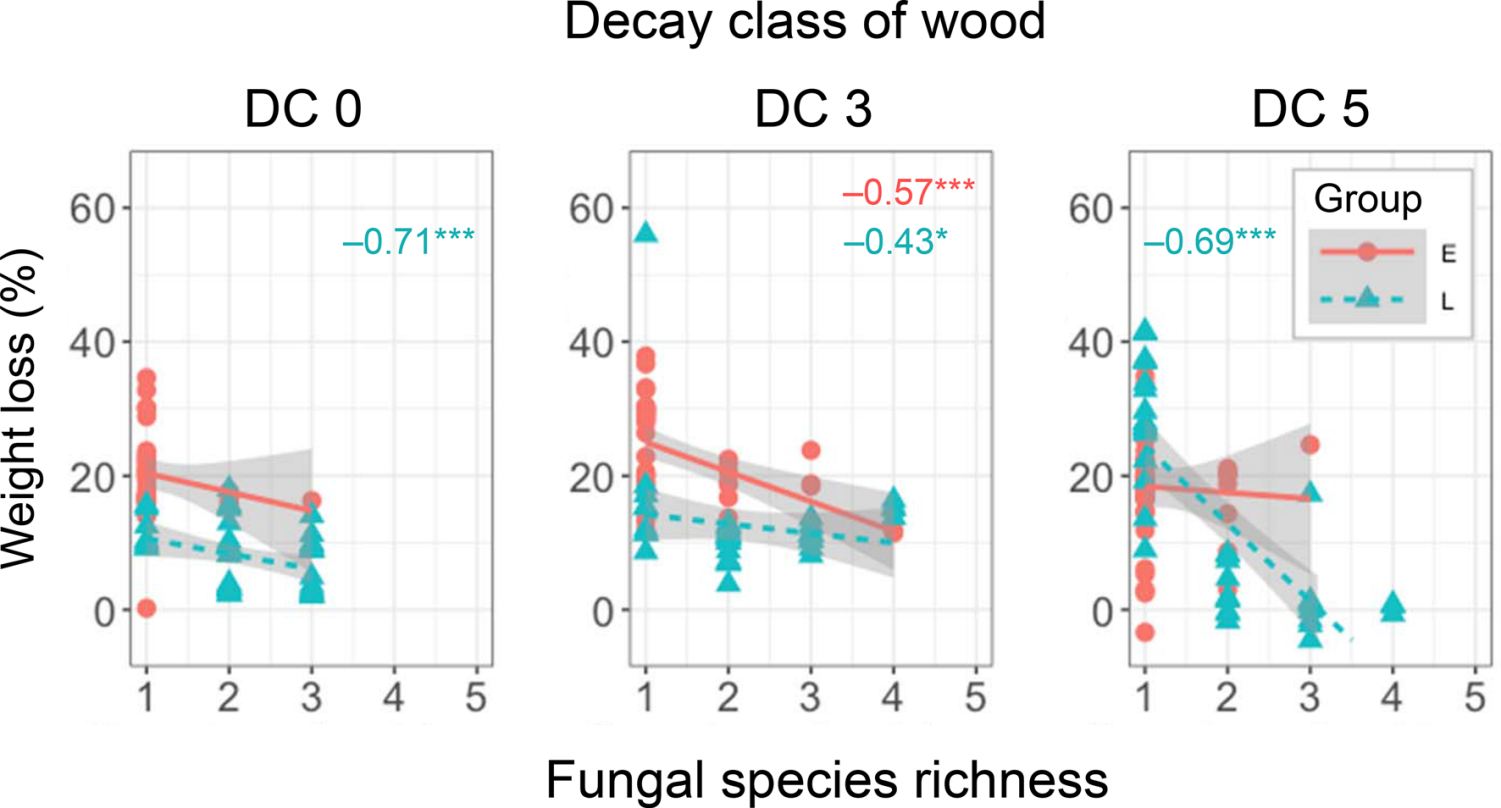
Relationships between fungal species richness (without contaminant OTUs) and wood weight loss (% of control wood). Red round symbols indicate E group, and blue triangles L group. Red and blue numbers with asterisks represent significant Pearson’s correlation coefficients for groups E and L, respectively. Asterisks indicate *P < 0.05 and ***P < 0.001.

**Figure 6:**
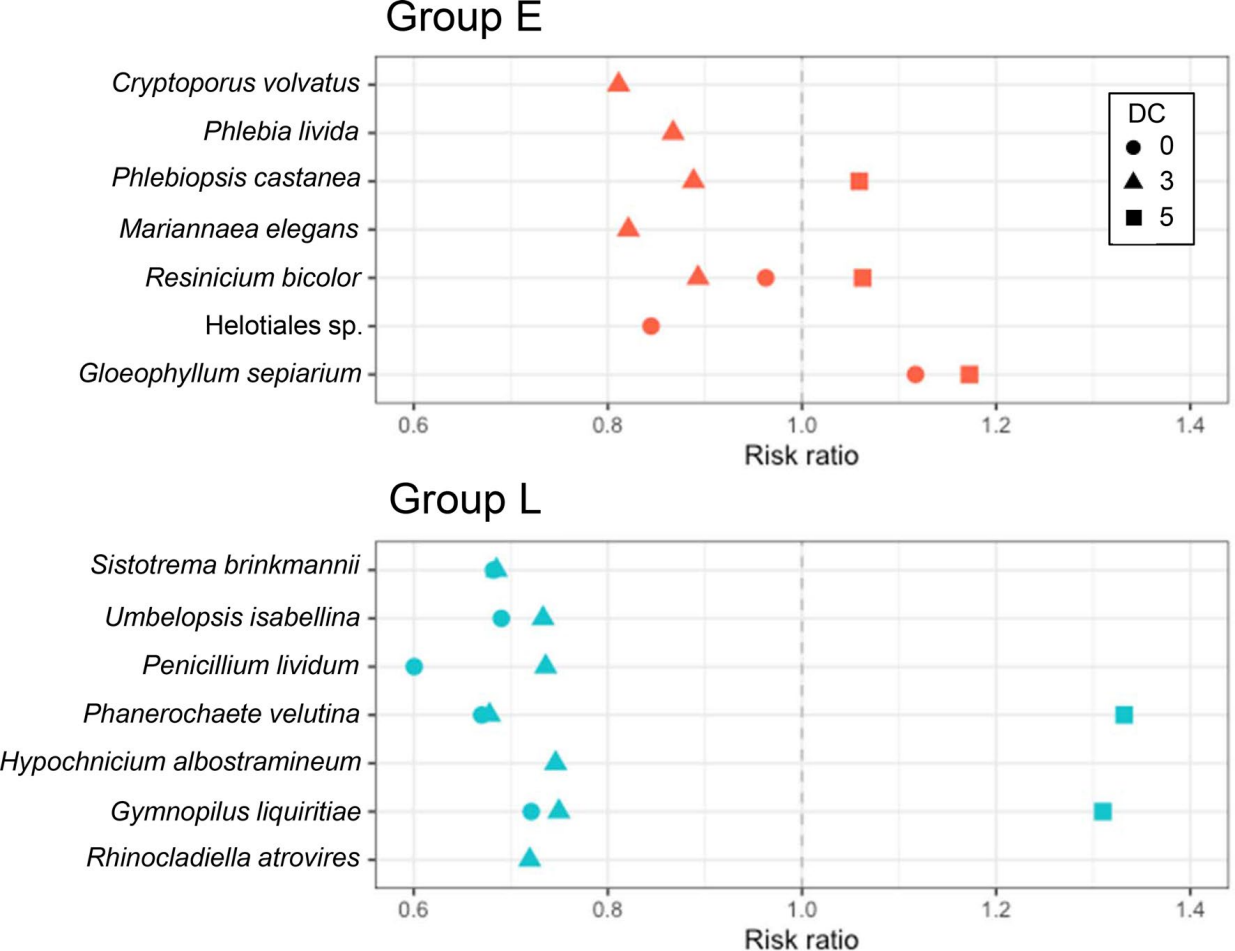
Risk ratios for effects of relative abundance (DNA read number) of fungal species detected after 5-month microcosm incubations on weight loss (% of control wood) of wood powder in decay classes (DC) 0, 3, and 5. Red and blue symbols indicate data from E and L groups, respectively. Ratios < 1 and > 1 indicate magnitude of negative and positive effects of fungal species on weight loss of wood powder, respectively.

The relationship between fungal species richness and wood decomposition confirmed that the two are indeed tightly linked (Fig. 5). Weight loss percentage relative to the control wood was negatively correlated with fungal species richness in fungal group E on DC 3 wood, and in fungal group L on wood of all decay classes. Generalised linear model results showed that the relative abundance of detected fungal species had a negative effect on wood weight loss in DC 0 and 3, except for *Gl. sepiarium* in DC 0, but a positive effect in DC 5 (Fig. 6). Likewise, *Ph. castanea* had a negative effect on wood weight loss in DC 3, but a positive effect in DC 5; *R. bicolor*, *Ph. velutina*, and *Gy. liquiritiae* had negative effects on wood weight loss in DC 0 and 3, but a positive effect in DC 5. The magnitude of negative and positive effects was stronger in groups L than group E.

## Discussion

Our results showed that the relationships between fungal species richness and wood powder weight loss were negative or neutral, which is in line with the results of previous microcosm studies^11,12^. The relationships were significant in group L fungal communities in all wood powder substrates tested, whereas significant relationships were recorded only for DC 3 wood powder in group E (Fig. 5), supporting our hypotheses about the effects of fungal community (hypothesis 1) and the decay class of the wood substrate (hypothesis 2) on fungal diversity–decay relationships. The literature suggests that a possible explanation for this negative relationship is a negative effect of competition stress on decomposition^12^. Antagonistic interactions between fungal mycelia force fungi to invest more in the production of secondary metabolites and enzymes necessary for competition than in those necessary for growth and decomposition^20,33,41–43^. This investment causes increased respiration^44^ and consequently reduces the CUE of the mycelia^29^.

The negative diversity–decomposition relationship in group E fungal communities was detected only in DC 3 wood powder (Fig. 5). Since the physical properties of the wood, such as density and water content, were normalised by pulverisation in the present study, the chemical properties of DC 3 wood––i.e. its delignified holocellulose content––might give rise to the negative diversity–decomposition relationships observed in DC 3 wood with group E fungi. We previously reported that the density of DC 3 sapwood was significantly decreased to nearly half that of DC 0 sapwood, while concentrations of acid-unhydrolysable residue (Klason lignin) and total carbohydrate (holocellulose) did not differ between these decay classes. This indicates simultaneous decomposition of lignin and holocellulose in DC 3 sapwood^40^. Therefore, lignin, which protects holocellulose in fresh sapwood, was mostly removed in DC 3 making holocellulose accessible for a variety of microorganisms that are not lignin decomposers^27^. Previous studies have reported that wood decomposed by the common white rot fungi *Trametes versicolor* and *Bjerkandera adusta* greatly improved wood hydrolysis with a commercial enzyme mixture^45,46^, although these fungi are simultaneous decomposers of lignin and holocellulose and not selective lignin decomposers^47^. Improved accessibility to holocellulose increased fungal species richness with wood decomposition^21,48–50^ and thus induced intense interspecific fungal competition compared to DC 0 wood, in which holocellulose was protected by lignin, and DC 5 wood, in which the holocellulose content was depleted^19,51^.

Interestingly, the relative abundances of detected fungal species displayed negative associations on wood weight loss in DC 0 and DC 3, but the reverse was true in DC 5 (Fig. 6). This difference is attributable to a combination of two mechanisms related to resource quality and fungal competition. The first is a change in the wood decay ability of the focal fungus because of difference in wood quality^27^. In both DC 3 and 5, *Ph. castanea* in group E dominated almost entirely (Supplementary Fig. S2a), although the effect of this fungus changed from negative in DC 3 to positive in DC 5 (Fig. 6). In this case, *Ph. castanea* was responsible for wood decomposition in both decay classes with little interaction with other species. The second mechanism is a change in the wood decay ability of a focal fungus because of interactions with other fungi. For example, *R. bicolor* in group E coexisted with *Ph. castanea* in DC 0 and with *Gl. sepiarium* and *Ph. livida* in DC 3, whereas it dominated alone in DC 5 (Supplementary Fig. S2a). Similarly, *Ph. velutina* in group L coexisted with *S. brinkmannii* in DC 0, but it dominated almost entirely in DC 3 and 5 (Supplementary Fig. S2b). The negative effects of these fungi in coexistence with other species on wood weight loss in DC 0 and 3 suggest that interactions with other fungal species retard wood decomposition. The effect of *Gy. liquiritiae* in group L differed between decay classes (Fig. 6) even though this species coexists with *S. brinkmannii* in DC 0 and 3 and with many other species in DC 5 (Supplementary Fig. S2b). We speculate that differences in species association may cause these results, because the relationship between *Gy. liquiritiae* and *S. brinkmannii* was negative, while the relationships between *Gy. liquiritiae* and other fungal species in DC 5, such as *U. isabellina*, was positive (Fig. 3). More antagonistic (competitive) associations decrease the CUE of fungi compared to less competitive associations^52^.

The extent to which our laboratory results apply to natural ecosystem dynamics remains to be investigated. The negative relationships between fungal species richness and wood weight loss, particularly observed in the late-stage decay fungal community (group L), however, were not consistent with the results of van der Wal et al.^7^, who reported positive relationships between fungal species richness and wood weight loss in later decay stages in the field. Wood pulverisation may have some effects on fungal diversity–decomposition relationships, which is a limitation of the present study. However, previous experimental studies using intact wood pieces also showed negative relationships between fungal species richness and wood weight loss^11,12^. This discrepancy could be explained in three ways. First, the range of species richness in the present study and in previous laboratory studies^11,12^ was much smaller than that in the field data of van der Wal et al.^7^. It is quite common for hundreds of fungal OTUs to be detected in a single log in the field^7,22,53^. Negative diversity–weight loss relationships are known to saturate within < 10 fungal species in laboratory conditions^11^, and a more diverse fungal community may show increased decomposition due to factors other than competition, such as resource partitioning and facilitation. However, testing the effects of high fungal diversity on decomposition in a small-scale microcosm system is difficult because the smaller the system, the larger the number of species that are competitively excluded. A smaller number of species is likely to survive during the experimental period due to the low carrying capacity of the system, as in the classical theory of island biogeography^54^. In the present study, fungal species richness after 5 months of competition was 1–4 species, even though we started the experiment with 11–12 species. Similarly, Fukami et al.^12^ detected only five species after 12 months of incubation of a wood microcosm that began with 10 fungal species. Testing the effect of resource volume on diversity–decomposition relationships would require an enlarged microcosm system.

The second possible reason for the discrepancy between laboratory and field studies is the fluctuation of environmental conditions in the field. In contrast to the stable conditions in the laboratory, environmental factors such as temperature and moisture fluctuate daily and seasonally in the field. The insurance hypothesis^55^ predicts that biodiversity protects ecosystems from functional decline because larger higher species richness provides a greater guarantee that some species will maintain function even if others fail in certain environmental conditions. Different fungal species have their own ranges of abiotic stress tolerance^56^, and communities with high diversity can cope with environmental fluctuations and maintain stable functionality^57^. Toljander et al.^11^ reported that environmental fluctuation facilitates species coexistence and increases decomposition in wood decay fungal communities in microcosm systems.

The third possible reason for high fungal species richness being associated with greater decay rate in the field^7^ is that observed species richness in the field is not a cause but a result of progressive wood decomposition. Even if the decomposition started at the same time point, variations in abiotic and biotic conditions could easily cause variations in the progress of wood decomposition^1^, even within the same log^58^. Additionally, fungal species richness generally increases along with wood decomposition^21,22,48,50^. Microcosms forcing diversity losses, as in the present study, may be an indicator that patterns of species richness recorded in wood decay studies in the field are the result of significant death/loss and replacement by soil/airborne colonisers.

In the present study, several fungal OTUs that were not inoculated into the microcosm were detected by DNA metabarcoding, although we did not observe any visible hyphal growth on control samples. All identified OTUs among those ‘contaminated’ OTUs were well-known wood-inhabiting fungi^50^. However, instead of their dominance in control samples, the weight loss of control samples over six months of incubation was less than 6%, which is in the range of the weight loss of control wood powder in a similar experiment that used powder that had been completely sterilised by autoclaving. This small loss is probably due to leaching of labile components from the wood^27^. These results suggest that the contaminant fungal OTUs in the present study were from relic DNA of wild fungal communities in the sample^59^. Nevertheless, using other sterilisation methods, such as autoclaving and gamma irradiation, may provide more clarity in future research.

## Conclusions

We evaluated the effects of fungal communities and substrate quality on the relationships between fungal species richness and wood weight loss in microcosms with early and late fungal communities with known fungal succession and wood powder substrates in three stages of decay. The results were in line with previous laboratory studies that reported negative relationships between fungal species richness and substrate weight loss. The novel finding of our study is that fungal communities in later stages of wood decomposition showed clearer negative associations with decay rate than communities in earlier stages. The quality of the wood powder substrate also affected fungal diversity–decomposition relationships, as the negative relationships observed in the early-decay communities was significant only with wood powder substrate of the intermediate decay stage. Nevertheless, the negative fungal diversity–decomposition relationships observed in the present study and in previous laboratory studies^11,12^ are not consistent with the positive relationships observed in previous field studies^7^. Therefore, further investigation is necessary to clarify the effects of fluctuations in environmental conditions^11^ and substrate volumes^32^ on diversity–decomposition relationships, and it is crucial to bridge the gap between laboratory and field studies. Species diversity and substrate quality affect each other in a feedback loop that in turn affects decomposition^23^. We propose that more reliable prediction and management of carbon dynamics in forest ecosystems requires greater attention to such feedback loops and specifically to the interactive effects of species diversity and substrate quality on decomposition.

## Methods

### Fungal cultures

Pure cultures of 74 fungal species were isolated on malt extract agar (MA; 2% malt extract, 1.5% agar, w v^–1^; Nakalai Tesque, Kyoto, Japan) from a total of 375 wood chips (ca. 2 mm^3^ for each chip, taken from both sapwood and heartwood) cut out from 5 living and 20 standing dead snags of *Pinus densiflora* (diameter at breast height ranged from 11 to 34 cm) in a *P. densiflora* plantation at the Kawatabi Field Science Center of Tohoku University (38°46′N, 140°45′E, 547 m a.s.l.), Miyagi, Japan^40^. Sixteen of the 74 fungal species were selected for experimental use, along with an additional seven species isolated from fruiting bodies on *P. densiflora* deadwood, including some basidiomycete species commonly recorded from *P. densiflora* deadwood at other sites in Japan^38,60^. Thus, 23 species were used in the experiments (Table 1), based on occurrence frequency and feasibility of culture. Fungal assemblages were separated into two groups, E and L. Group E consisted of 11 early coloniser species and group L consisted of 12 late coloniser species, consistent with natural successional order observed in previous studies^38–40^. Group E consisted of eight species in Basidiomycota and three in Ascomycota; Group L consisted of eight species in Basidiomycota, three in Ascomycota, and one in Mucoromycota. We included a strain of Mucoromycota in this experiment because fungi in this group are commonly detected in deadwood, particularly in the late stages of decomposition^22,50,53^. Fungi in Mucoromycota are generally weak decomposers of wood structural components^27,61^. However, they can accelerate decomposition by other fungal species by utilising low-molecular sugars produced by the other species, because the accumulation of such sugars reduces cellulase production by fungal decomposers^62^. Groups E and L both included white rot and brown rot basidiomycetes (six white rot fungi and two brown rot fungi in group E; seven white rot fungi and one brown rot fungus in group L). All cultures except for *Trichoderma harzianum* were deposited in the culture collection of the NITE Biological Resource Center (NBRC, Chiba, Japan). Sequence data for internal transcribed spacer (ITS) regions of rDNA were deposited in the DNA data bank of Japan (Table 1).

### Microcosms

Sapwood particles were obtained from six samples of *P. densiflora* deadwood collected at the study site described above. Three samples were in DC 3 (moderately decayed) and the others were in DC 5 (well decayed) using a five-level decay classification system^63^. Further, fresh (non-decayed, DC 0) sapwood particles were obtained from 1.3 m above the ground from the trunks of three freshly felled *P. densiflora* trees. The diameters of the sampled deadwood specimens were 10–35 cm.

Three sapwood samples in each of the three decay classes (DC 0, DC 3, DC 5) were crushed until they passed through a 2 mm mesh using a Retsch SM 300 cutting mill (Verder Scientific Co. Ltd., Germany) and mixed well for use as decomposition substrate. The chemical properties of the wood powder are shown in Supplementary Table S2. The sapwood in DC 5 contained ca. 1.3 times higher concentrations of acid-unhydrolysable residue (Klason lignin), ca. 1.3 times lower concentrations of total carbohydrate (mainly holocellulose) and 3–4 times higher concentrations of nitrogen than wood in both DC 0 and DC 3. The contents of glucosamine hydrolysed from chitin in fungal cell walls were less than 1.5% in all decay classes.

Next, 3 g of air-dried wood powder was packed into a polyester teabag (Tokiwa Kogyo, Japan), sealed, sterilised with ethylene oxide gas at 55 °C for 4 h (2 h × 2), and aseptically placed onto a plain agar plate in a 9 cm petri dish (2 cm depth). The mesh of the teabag was fine enough to prevent leakage of wood powder.

### Fungal inoculation

One of the 20 species (10 from group E and 10 from group L) was selected as an initial species and was inoculated 1 month earlier than successor species (10 species in group E and 11 species in group L), to induce variety in species diversity through the priority effect by incubation with the same set of species after the incubation period^12^. From colonies growing on MA plates of each fungal species, 5 mm diameter plugs were cut out using a sterilised cork borer and one plug of each initial species (Table 1) was placed beside each teabag (Fig. 1). Inoculations of successor species were performed in the same manner one month after the inoculation of the initial species. The relative locations of species inoculation points were held constant across treatments (Fig. 1). Sixty treatments in a factorial design (3 decay classes × 2 groups of fungi × 10 initial species) were replicated five times. Five control samples (inoculated with sterile agar plugs) were prepared for each decay class of wood substrate. Throughout the experiment, dishes were sealed with Parafilm and stored in the dark at 25 °C.

### Decomposition measurement

Five months after the inoculation of successor species, wood powders were harvested and freeze-dried to constant weight. The percentage of wood weight loss was calculated for each teabag as the ratio of dry weight at harvest to initial dry weight or to the dry weight of control wood samples inoculated with sterile agar plugs.

### Species occurrence

Fungal community composition in 300 harvested wood powder samples (and 15 control samples) was determined by DNA metabarcoding. The freeze-dried wood powder was ground and homogenised with a Multi-beads shocker (Yasui Kikai, Osaka, Japan). DNA was extracted from 50 mg of homogenised sample using an MoBio PowerPlant Pro Kit (QIAGEN, Venlo, Netherlands) following the manufacturer’s instructions. The DNA concentrations of extracted samples were checked using Qubit (Thermo Fisher, Tokyo). Five samples without sufficient DNA were removed from the following analyses. The fungal ITS1 gene region was amplified by two-step PCR with ITS1F_KYO1/ITS2_KYO2 primers^64^ containing tails for adding indices and Illumina flow cell adapters in a second amplification. *Ex Taq* Hot Start Version (Takara Bio, Kusatsu, Japan) was used in both PCR steps. Sequencing was performed on a MiSeq with a 600 cycle v3 kit (Illumina, San Diego, CA, USA). The details of the PCR reaction for sample preparation for MiSeq sequencing are given in Supplementary methods.

All sequence reads obtained from MiSeq sequencing were filtered to leave 513,711 reads after removing low-quality and chimeric sequences. The chimera check was carried out with Claident v0.2.2017.05.22 software^65^ using the UNITE database (https://unite.ut.ee, accessed 1st July 2018). Quality-filtered sequences were classified into molecular OTUs and taxonomically identified using the Claident software original fungi-ITS database, which is structured based on the International Nucleotide Sequence Database (INSD, http://www.insdc.org, accessed 3rd December 2019). The identification threshold of sequence similarity was 97%, which is widely used for the fungal ITS region^66^. OTUs not identified to the genus level by Claident were further checked with NCBI blastn and GenomeMatcher ver. 2.205^67^ using ITS sequences of the fungal strains as references (DDBJ accession numbers are shown in Table 1). For each sample, OTUs with less than 5% of the total reads were removed. We took our unrarefied OTU table and conducted 200 rarefactions for each sample at a depth of 100 sequence reads since detected OTU numbers were saturated in all samples at this depth after filtering (Supplementary Fig. S3); 27 samples with < 100 reads were removed from the analyses. After rarefaction, OTUs with 0 reads were removed from the analyses. This process accounted for sequence length across individual samples and let us compare the read number as a measure of the relative abundance of OTUs. It is necessary to take care to use sequence read percentage as a measure of relative abundance because of possible sequencing bias, particularly for environmental DNA samples that consist of DNA from a wide phylogenetic range^10^. However, in the present study, the problem is much smaller than with eDNA samples because the communities mainly consisted of known sets of fungal species.

Wood substrates used in the present study were collected from the field and thus contained native fungal communities. Although the wood was sterilised using ethylene oxide gas before the experiment, this likely did not destroy all fungal DNA; not a small number of detected OTUs were fungal species not used for inoculation (Supplementary Table S1). Also, some fungi that were used for inoculation were detected from samples in which they were not inoculated, e.g. fungi in group E were detected from wood inoculated with group L species and vice versa. However, as shown in the result of the present study, such DNA was not associated with wood weight loss and might be remnant DNA from wild fungal communities that were no longer active at the time of our experiments. We employed strong filtering as described above to remove OTUs of this remnant DNA as much as possible, and thus reduced the fungal OTU number from 348 to 24. Although huge numbers of OTUs were removed, we confirmed that OTUs of inoculated fungi were not excluded. We are not able to distinguish remnant DNA of a particular fungus from DNA of the same fungus in a sample in which that species was inoculated. However, such cases must be rare and the effects on our results negligible since we found only six cases of minimal relative abundances where fungi in one group were detected from wood inoculated with the other group.

### Statistical analysis

The effects of initial species and the decay class of the wood substrate on fungal species richness, the relative abundance of each fungal species, and wood weight loss were assessed using analysis of variance (ANOVA), with initial species, decay class of wood substrate, and their interactions as independent variables and fungal species richness, the relative abundance of each fungal species, and wood weight loss as dependent variables. Tukey-HSD posthoc multiple comparisons were used with Bonferroni adjustment of probability values.

Hierarchical modelling of species communities (HMSC) was performed on each experiment inoculated with fungal group E and L to assess association networks of fungal species^68^. We used relative abundance data on fungal species and fitted a HMSC model to data with Bayesian inference by running 150,000 Markov Chain Monte Carlo iterations with a thinning factor of 100. The decay class of the wood substrate was set as a random effect. We assessed the residual correlation between fungal OTUs after accounting for the covariate effects to determine whether fungal species respond to one another or to unmeasured differences related to substrate decay class.

Pearson’s correlation coefficient between fungal species richness and wood weight loss was calculated for fungal groups E and L on woods of DC 0, 3, and 5. The effect of the relative abundance of inoculated fungal species on wood weight loss was evaluated using generalised linear models for groups E and L separately for each wood decay class (six models in total). Gaussian distribution was assumed and an identity link function was used in the models. The relative abundances of all detected fungal species were set as fixed variables in the null model, and the best model was selected based on the Akaike’s information criterion by backwards stepwise selection of fungal species. Coefficients of the best models were exponentiated to obtain risk ratios. Ratios > 1 indicate that the explanatory variables had a positive effect on wood weight loss, while ratios < 1 indicate negative effects. Differences from 1 indicate the magnitudes of effects. The level of collinearity between predictor variables was checked by calculating the variance inflation factor (VIF); all VIF value were < 7, indicating low levels of multicollinearity in the models. All statistical analyses were conducted using R ver. 3.6.1^69^.

## Supplementary Information

Supplementary Information

## Data Availability

The datasets generated during the current study are available from the corresponding author on reasonable request.
