# Supplementary material for: Decay stages of wood and associated fungal communities characterise diversity–decomposition relationships
Source: Sci Rep. 2021 Apr 26;11:8972. doi: 10.1038/s41598-021-88580-2 (PMC8076174; doi:10.1038/s41598-021-88580-2)
Supplement: Supplementary file 1 — Supplementary Information [file 41598_2021_88580_MOESM1_ESM.pdf]

Supplementary materials

Decay stage of wood and associated fungal communities characterize diversity–decomposition relationships

Yu Fukasawa[1], Kimiyo Matsukura[2]

[1] Graduate School of Agricultural Science, Tohoku University, 232-3 Yomogida, Naruko, Osaki, Miyagi 989-6711, Japan
[2] Sado Island Center for Ecological Sustainability, Niigata University, 94-2 Koda, Sado, Niigata 952-2206, Japan

[*]Corresponding author: yu.fukasawa.d3@tohoku.ac.jp

Table S1 Fungal operational taxonomic units (OTUs) detected after 6 months incubation period.

OTU number	Taxa	Group
OTU_12	*Phlebiopsis castanea*	E
OTU_13	*Rhinocladiella atrovirens*	L
OTU_18	*Hypochnicium albostramineum*	L
OTU_62	*Femsjonia uniseptata*	–
OTU_65	Rhytismatales	–
OTU_68	*Cryptoporus volvatus*	E
OTU_83	Ascomycota	–
OTU_101	*Mariannaea elegans*	E
OTU_138	*Scytalidium* sp.	–
OTU_156	*Saitozyma podzolica*	–
OTU_158	*Phlebia livida*	E
OTU_159	*Gymnopilus liquiritiae*	L
OTU_201	Fungi	–
OTU_219	*Phanerochaete velutina*	L
OTU_237	*Gloeophyllum sepiarium*	E
OTU_241	Helotiales sp.	E
OTU_251	Fungi	–
OTU_255	*Penicillium lividum*	L
OTU_272	*Umbelopsis* sp.	–
OTU_288	*Resinicium bicolor*	E
OTU_304	*Umbelopsis isabellina*	L
OTU_307	*Sistotrema brinkmannii*	L
OTU_312	Ascomycota sp.	–
OTU_314	Dikarya	–

–, not inoculated in the present study.

Table S2 Contents of acid-unhydrolyzable residue (AUR, Klason lignin), total carbohydrate (TCH), glucosamine, total nitrogen and total carbon in *Pinus densiflora* sapwood powder used in the microcosm experiment.

	DC 0 (n=5)*	DC 3 (n=5)	DC 5 (n=5)
AUR (%)	29.4±1.0	30.6±2.2	39.0±3.1
TCH (%)	67.8±1.4	64.9±2.4	52.2±1.3
Glucosamine (%)	1.5±0.1	1.1±0.0	1.0±0.0
Nitrogen (%)	0.059±0.005	0.086±0.005	0.301±0.007
Carbon (%)	47.3±0.1	47.1±0.1	48.5±0.2

Mean±SD.

DC, decay class

* n=4 for AUR, TCH, and glucosamine.

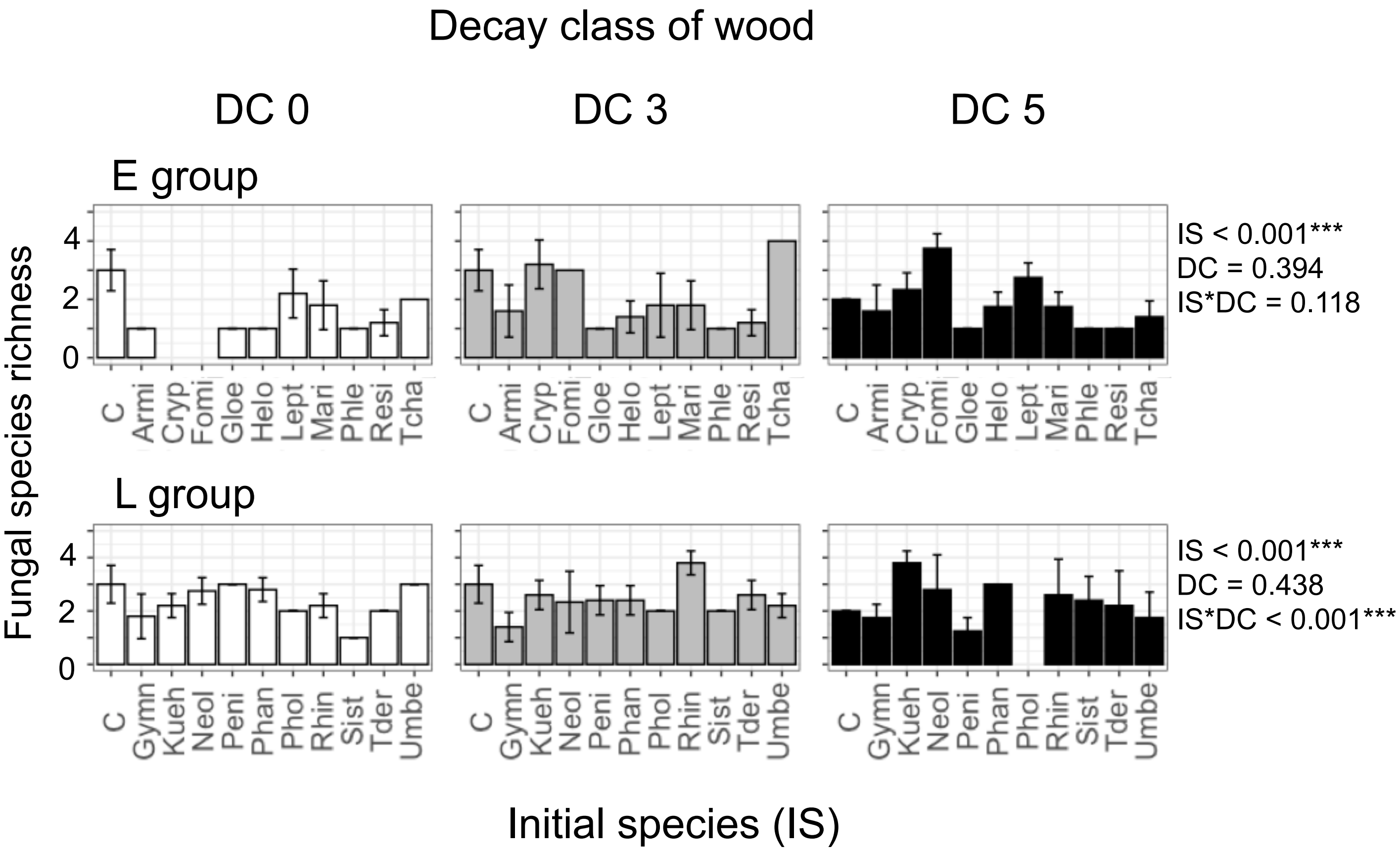

Fig. S1 Effects of fungal colonization history on fungal species richness including contaminant fungal OTUs. Species richness (mean ± SD) was measured 5 months after all species were introduced. *P* values from ANOVAs are provided for effects of initial species (IS), decay class (DC), and their interaction (IS*DC). Asterisks indicate *** $P < 0.001$.

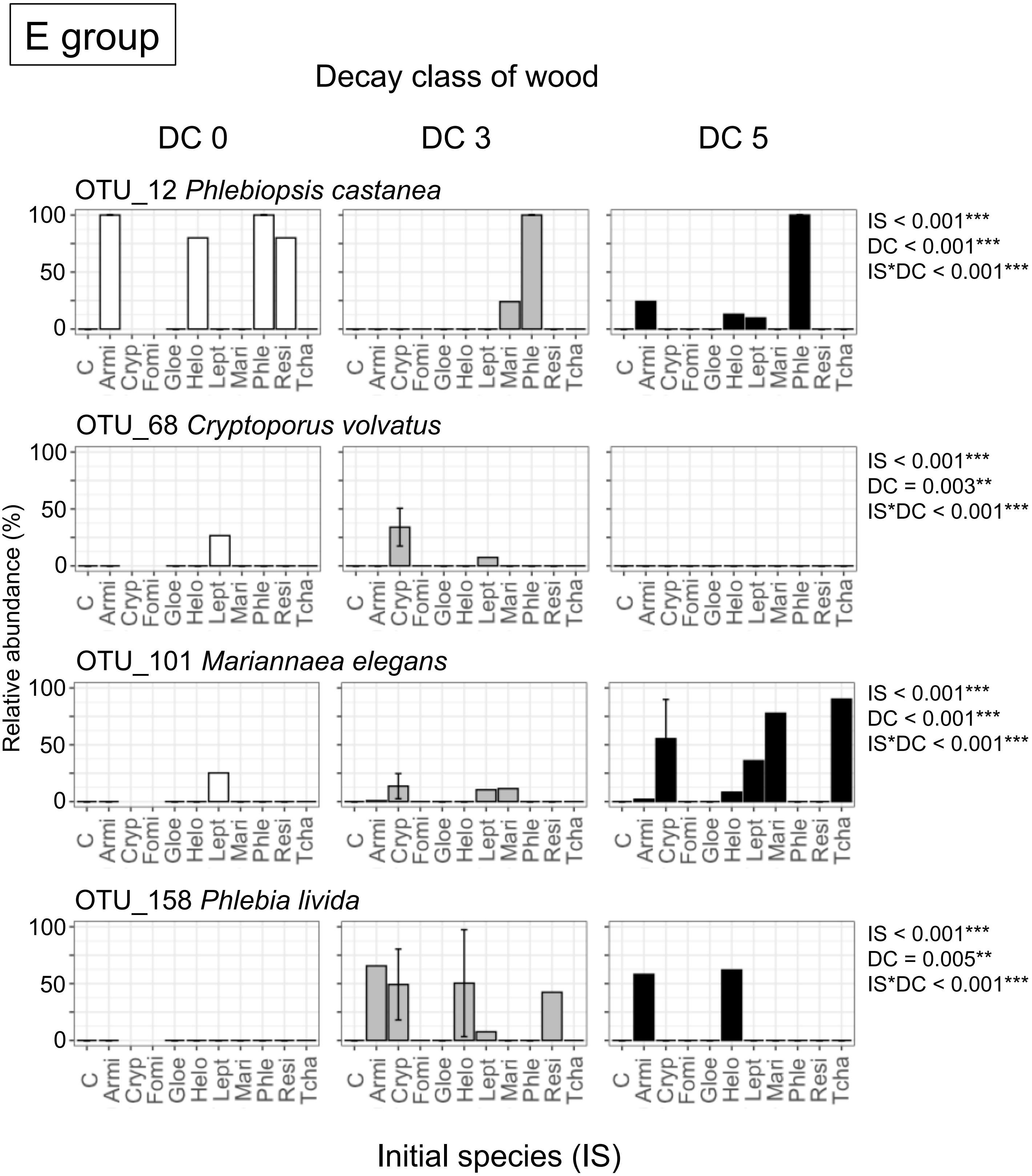

Fig. S2a Effect of fungal colonization history on relative abundance (sequence reads) of fungal OTUs in E group, measured 5 months after all species were introduced. *P* values from ANOVAs are provided for effects of initial species (IS), decay class (DC), and their interaction (IS*DC). Asterisks indicate ** $P < 0.01$ and *** $P < 0.001$.

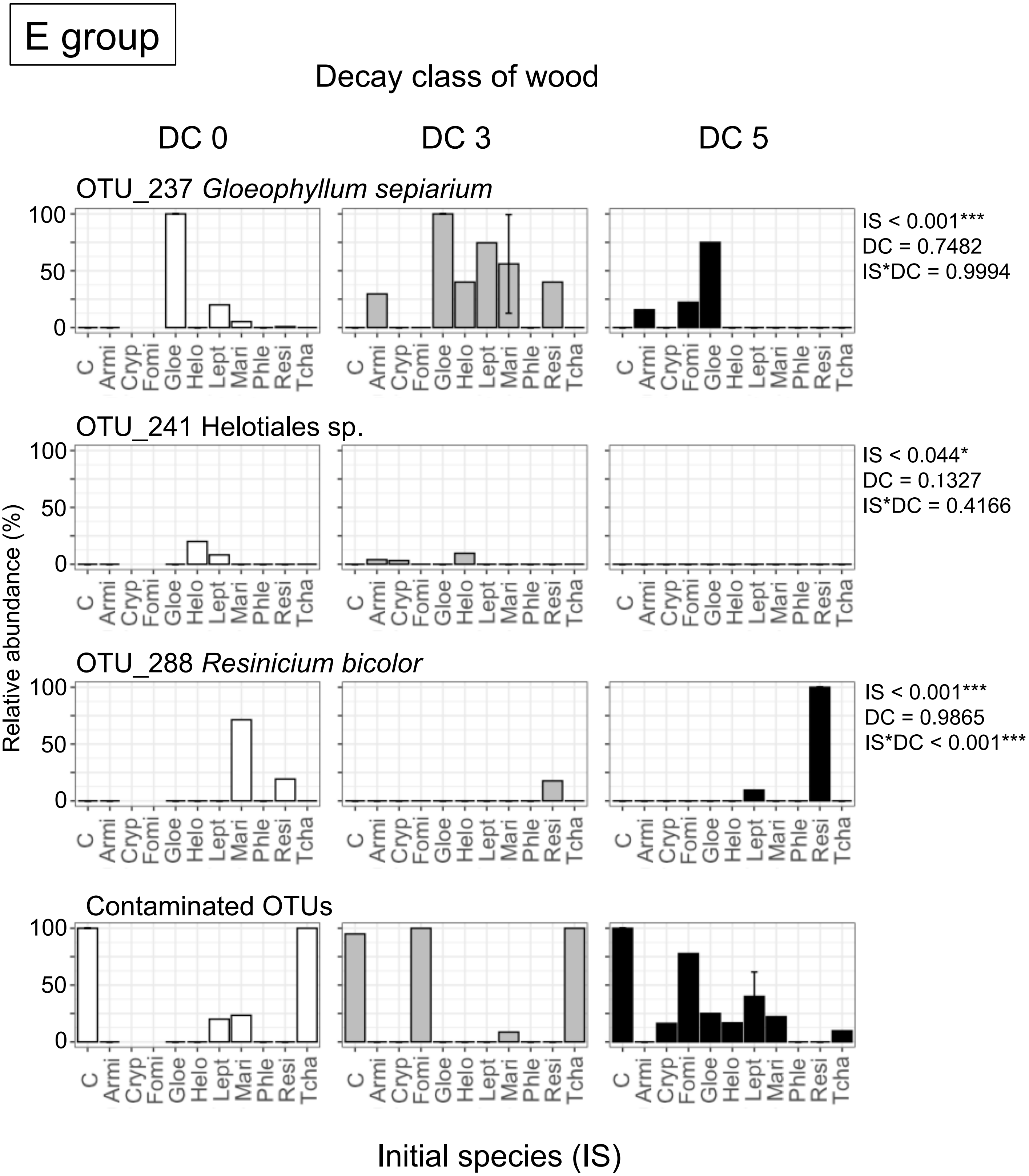

Fig. S2a_*Continued*. Effect of fungal colonization history on relative abundance (sequence reads) of fungal OTUs in E group, measured 5 months after all species were introduced. *P* values from ANOVAs are provided for effects of initial species (IS), decay class (DC), and their interaction (IS*DC). Asterisks indicate * $P < 0.05$, ** $P < 0.01$ and *** $P < 0.001$.

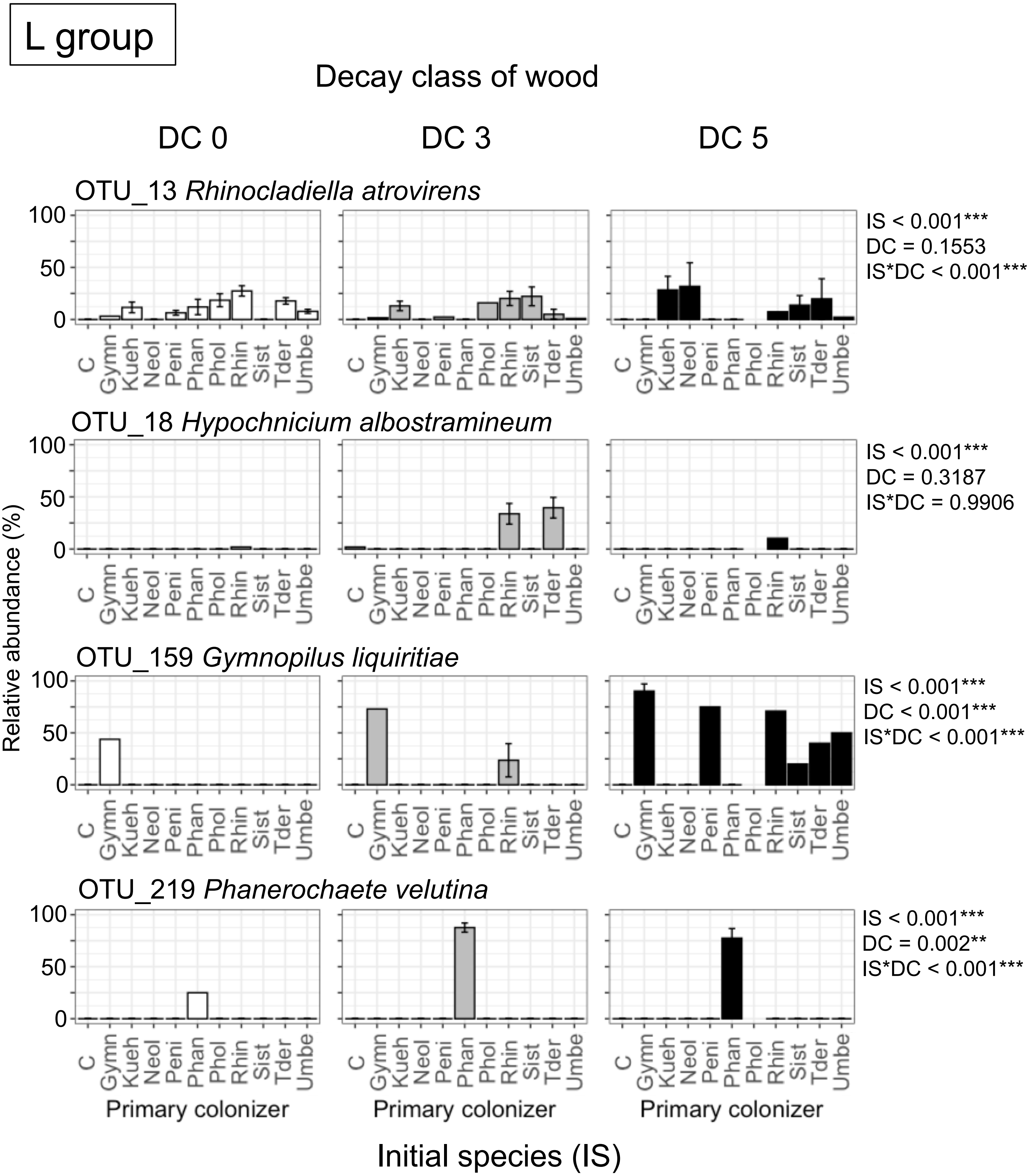

Fig. S2b Effect of fungal colonization history on relative abundance (sequence reads) of fungal OTUs in L group, measured 5 months after all species were introduced. *P* values from ANOVAs are provided for effects of initial species (IS), decay class (DC), and their interaction (IS*DC). Asterisks indicate ** $P < 0.01$ and *** $P < 0.001$.

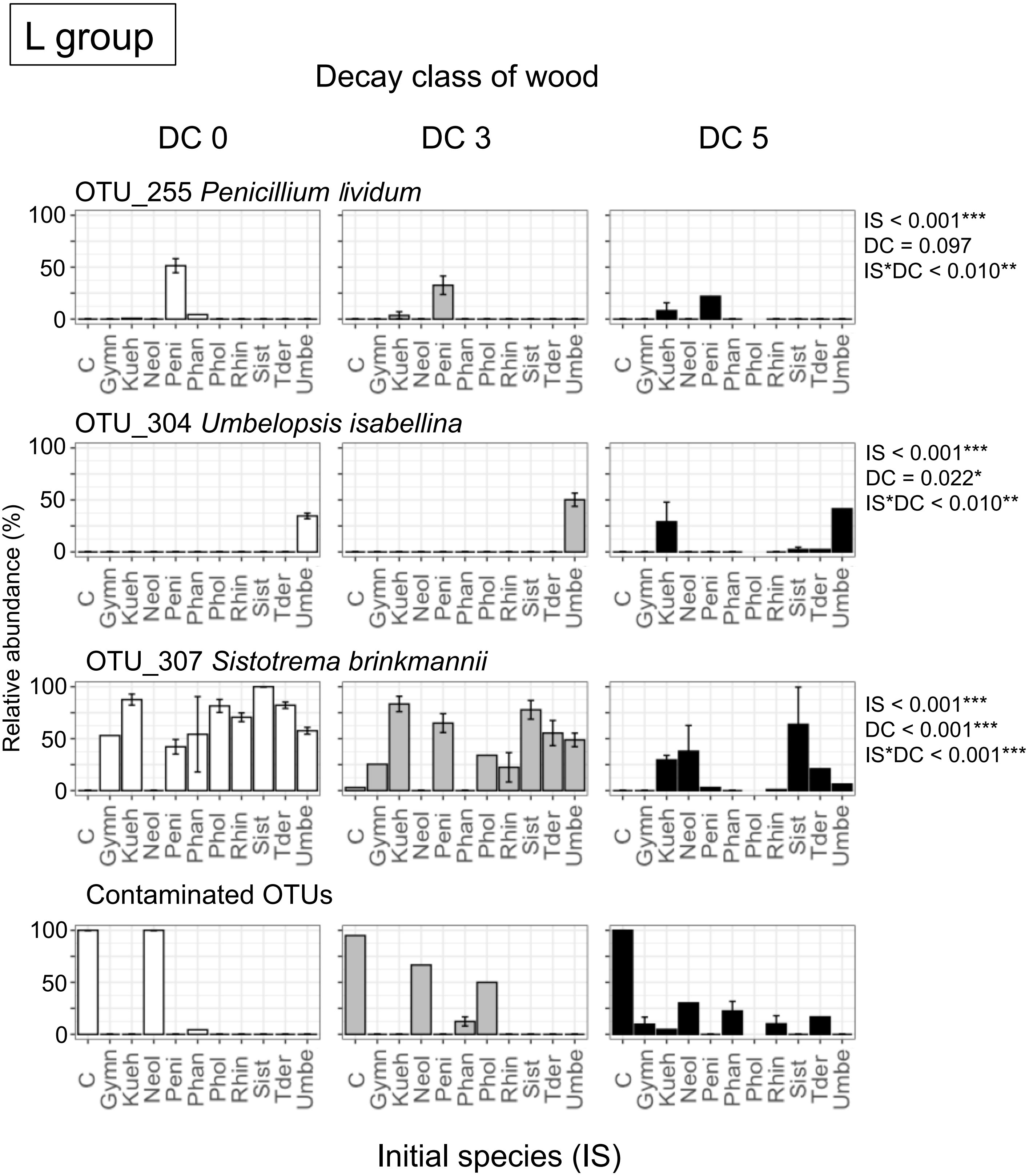

Fig. S2b_*Continued*. Effect of fungal colonization history on relative abundance (sequence reads) of fungal OTUs in L group, measured 5 months after all species were introduced. *P* values from ANOVAs are provided for effects of initial species (IS), decay class (DC), and their interaction (IS*DC). Asterisks indicate * $P < 0.05$, ** $P < 0.01$ and *** $P < 0.001$.

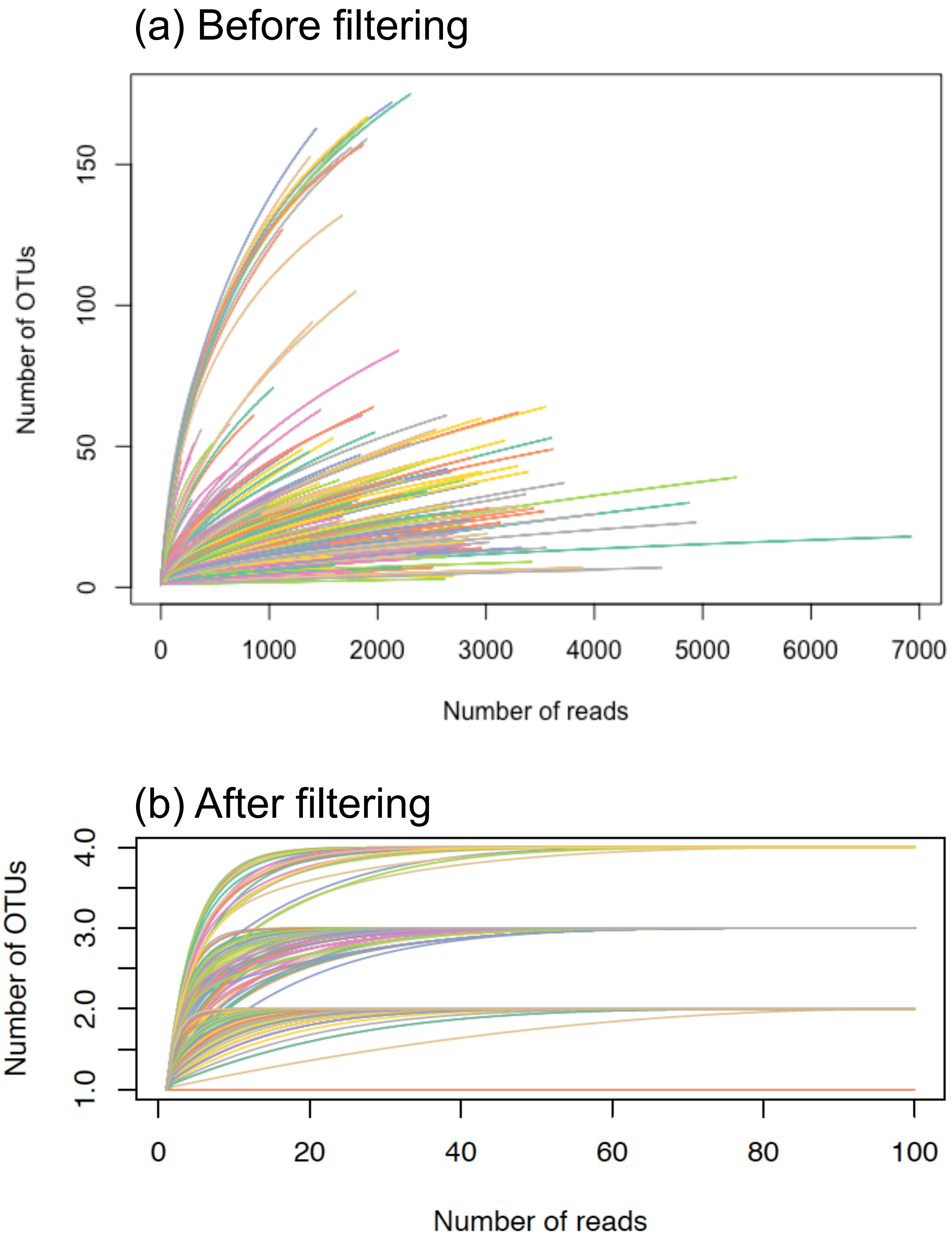

Fig. S3 Rarefaction curves for each sample before (a) and after (b) the filtering process.

Supplementary Methods

First PCR

The fungal ITS1 gene region was amplified using a two-step PCR protocol with ITS1F_KYO1/ITS2_KYO2 primers (Toju et al. 2012) in primary amplification containing tails for adding indices and Illumina flow cell adapters in a secondary amplification.

1st Forward primer (ITS1F_KYO1):

ACACTCTTTCCCTACACGACGCTCTTCCGATCT**CTHGGTCATTTAGAGGAASTAA**

1st Reverse primer (ITS2_KYO2):

GTGACTGGAGTTCAGACGTGTGCTCTTCCGATCT**TTYRCTRCGTTCTTCATC**

Where the left parts of the sequence shown in normal font are adapters to attach to the second PCR primers, and the right parts shown in bold font are specific sequences to the target ITS1 region.

The primary amplification was conducted using 0.5 µl template DNA, 6.6 µl nuclease-free water, 1.0 µl 10× *Ex Taq* buffer, 0.1 µl *Ex Taq* Hot Start version (Takara Bio, Kusatsu, Japan), 0.8 µl 2.5 mM dNTP mixture, 0.5 µl forward primer (10 µM), and 0.5 µl reverse primer (10 µM). A PCR cycling protocol was conducted with an initial incubation at 94°C for 4 min, 30 cycles of denaturation at 94°C for 30 s, annealing at 50°C for 30 s, and extension at 72°C for 1 min, and a final elongation step at 72°C for 10 min.

The amplicons from the first PCR were diluted 1:30 in sterile, nuclease-free water, and a second PCR reaction was set up to add the Illumina flow cell adapters and indices.

Secondary amplification

The secondary amplification was performed using the following recipe: 2.0 µl template DNA, 12.2 µl nuclease-free water, 2.0 µl 10× *Ex Taq* buffer, 0.2 µl *Ex Taq* Hot Start version (Takara Bio), 1.6 µl 2.5 mM dNTP mixture, 2.0 µl forward primer and reverse primer with index mix (10 µM). Cycling conditions were 12 cycles of denaturation at 94°C for 2 min, annealing at 60°C for 30 s, and extension at 72°C for 60 s, and a final elongation step at 72°C for 5 min.

2nd Forward primer:

AATGATACGGCGACCACCGAGATCTACAC-index1-ACACTCTTTCCCTACACGACGC

2nd Reverse primer:
CAAGCAGAAGACGGCATACGAGAT-index2-GTGACTGGAGTTCAGACGTGTG

The concentrations of each second PCR product (libraries) were measured using a Microchip Electrophoresis System (MultiNA, Shimadzu, Kyoto, Japan) with a DNA-2500 Reagent Kit (Shimadzu). The libraries from each sample, each with a different index, were then pooled in equimolar concentrations. To reduce the salt concentration, the mixed libraries were purified and the buffer was replaced with elution buffer by using a QIAquick PCR Purification Kit (Qiagen Sciences, Valencia, USA). Fragments in the size range of 200–650 bp in the purified library were isolated using the Pippin Prep DNA size selection system (Sage Science, Beverly, MA, USA). The final concentration was measured using a SYBR green quantitative PCR assay (Library Quantification kit; Clontech Laboratories, Mountain View, CA, USA) with primers specific to the Illumina system. The DNA library was diluted to 100×by nuclease-free water, and then further diluted to 1000×, 2000× and 4000× by Easy Dilution Buffer (Library Quantification Kit; Takara Bio). Concentrations of the standard DNA in the kit were 10 pM, 1 pM, 0.1 pM and 0.01 pM. Quantitative PCR was performed with 2.0 µl diluted DNA (or 2.0 µl standard DNA), 4.0 µl nuclease-free water, 4.0 µl 5× Primer mix, 10.0 µl Terra PCR Direct SYBR Premix (Library Quantification Kit; Takara Bio). All library dilutions and standard DNA were replicated in three wells. A PCR cycling protocol was conducted with an initial incubation at 98°C for 2 min followed by 25 cycles of denaturation at 98°C for 10 s, annealing at 60°C for 15 s, and extension at 68°C for 45 s. The PCR products were stored at 4°C. Data quality was checked using the KAPA pPCR Efficiency Calculator (Kapa Biosystems), and the DNA concentration of the pooled library was calculated with a standard curve. The final products were sequenced by an Illumina MiSeq sequencer (Illumina, San Diego, CA, USA) using a MiSeq 600 cycle v3 kit (Illumina) at Laboratory of Forest Ecology, Tohoku University.

References

Toju, H., Tanabe, A.S., Yamamoto, S., Sato, H. 2012. High-coverage ITS primers for the DNA-based identification of ascomycetes and basidiomycetes in environmental samples. *Plos One* **7**: e40863.
